# Effects of Female Sex Hormones on Susceptibility to HSV-2 in Vaginal Cells Grown in Air-Liquid Interface

**DOI:** 10.3390/v8090241

**Published:** 2016-08-30

**Authors:** Yung Lee, Sara E. Dizzell, Vivian Leung, Aisha Nazli, Muhammad A. Zahoor, Raina N. Fichorova, Charu Kaushic

**Affiliations:** 1 McMaster Immunology Research Centre, Michael G. DeGroote Centre for Learning and Discovery, McMaster University, Hamilton, ON L8S 4K1, Canada; leey27@mcmaster.ca (Y.L.); dizzels@mcmaster.ca (S.E.D.); vwleung@hsph.harvard.edu (V.L.); nazliaisha@gmail.com (A.N.); zahoorma@mcmaster.ca (M.A.Z.); 2 Department of Pathology and Molecular Medicine, McMaster University, Hamilton, ON L8S 4K1, Canada; 3 Department of Obstetrics, Gynecology and Reproductive Biology, Brigham and Women’s Hospital, Boston, MA 02115, USA; rfichorova@rics.bwh.harvard.edu; 4 Harvard Medical School, Harvard University, Boston, MA 02115, USA

**Keywords:** herpes simplex virus type 2, HSV-2, female genital tract, vaginal cells, air-liquid interface, female sex hormones, estradiol, progesterone, medroxyprogesterone acetate, Vk2/E6E7 cells

## Abstract

The lower female reproductive tract (FRT) is comprised of the cervix and vagina, surfaces that are continuously exposed to a variety of commensal and pathogenic organisms. Sexually transmitted viruses, such as herpes simplex virus type 2 (HSV-2), have to traverse the mucosal epithelial lining of the FRT to establish infection. The majority of current culture systems that model the host-pathogen interactions in the mucosal epithelium have limitations in simulating physiological conditions as they employ a liquid-liquid interface (LLI), in which both apical and basolateral surfaces are submerged in growth medium. We designed the current study to simulate in vivo conditions by growing an immortalized vaginal epithelial cell line (Vk2/E6E7) in culture with an air-liquid interface (ALI) and examined the effects of female sex hormones on their growth, differentiation, and susceptibility to HSV-2 under these conditions, in comparison to LLI cultures. ALI conditions induced Vk2/E6E7 cells to grow into multi-layered cultures compared to the monolayers present in LLI conditions. Vk2 cells in ALI showed higher production of cytokeratin in the presence of estradiol (E2), compared to cells grown in progesterone (P4). Cells grown under ALI conditions were exposed to HSV-2-green fluorescent protein (GFP) and the highest infection and replication was observed in the presence of P4. Altogether, this study suggests that ALI cultures more closely simulate the in vivo conditions of the FRT compared to the conventional LLI cultures. Furthermore, under these conditions P4 was found to confer higher susceptibility to HSV-2 infection in vaginal cells. The vaginal ALI culture system offers a better alternative to study host-pathogen interactions.

## 1. Introduction

Approximately 417 million people (range: 274–678 million) worldwide are seropositive for herpes simplex virus type 2 (HSV-2) with an incidence of 19.2 million [1]. HSV-2 infection is widely prevalent around the world and lacks a definitive cure, making it a critical global concern. More females (267 million) are infected with HSV-2 than males (150 million), and data indicates that seroprevalence is higher in women (25.6%) compared to men (17.8%) in the USA [1,2]. While some studies have linked the higher infection rates in females to the greater surface area of the female reproductive tract (FRT) compared to the male reproductive tract, other studies suggest that other biological factors such as presence of female sex hormones contribute to the difference in infectivity and seroprevalence [3,4,5,6,7].

Transmission of HSV-2 in women occurs predominantly in the FRT through the direct infection of genital epithelium [8]. Epithelial cells of the FRT mucosa are the primary barriers against other sexually transmitted viruses, such as human immunodeficiency virus (HIV) and pathogenic bacteria [9,10,11]. Vaginal mucosal lining, which is made of stratified squamous epithelium, plays a critical role in reducing the risk of pathogenic invasion in the lower FRT and contributes to the maintenance of vaginal tissue homeostasis [12,13,14,15]. Despite these recognized functions, much remains to be learned about their interactions with commensal and pathogenic organisms in the FRT. Thus, an in vitro model that closely simulates the in vivo environment of the lower FRT is crucial to elucidate the complex interactions between sexually transmitted pathogens and host cells.

In order to simulate the interactions in the lower FRT more closely, it is vital that we understand its unique physiology. The in vivo environment of the FRT is mainly regulated by female sex hormones like estradiol (E2) and progesterone (P4) [4,16,17]. The immune system of the FRT has evolved to balance the processes of procreation and immune protection through the regulation of E2 and P4, two hormones that are produced cyclically by the ovaries during the menstrual cycle [15,16]. Many studies have elucidated that sex hormones can modify antiviral immune function and alter susceptibility to sexually transmitted viruses [5,18,19,20,21]. E2 is considered to be generally protective against sexually transmitted viral infections, while P4 and progestin-based hormonal contraceptives have been shown to increase susceptibility [5,18,19,20,21,22,23]. Studies have demonstrated that the presence of E2 can enhance CD4+ T cell antiviral immunity in the female FRT [24] and also offer greater protection against HSV-2 specific pathologies [5,21], while P4 leads to excessive genital inflammation and can reverse the effects of E2 in vivo [19]. It is also important to consider the effects of hormonal contraceptives in sexually transmitted virus acquisition. Medroxyprogesterone acetate (MPA), a first generation synthetic progestin used by over 100 million women worldwide, has been shown to play a role in enhancing HSV-2 infection in rodent and non-human primate studies [23,25]. MPA also enhances HIV uptake and transcytosis of HIV in primary genital epithelial cells [26].

Significant efforts have been invested in understanding and modeling the complex environment of the lower FRT. Earlier culture models employed the conventional liquid-liquid interface (LLI) approach by providing a growth medium to both apical and basolateral sides of cultures [27,28,29,30,31,32]. However, the vaginal epithelium in vivo draws nutrients only from its basolateral side, and is in contact with air and a limited volume of mucus and secretion, forming a strong physical and biochemical barrier on its apical side against the vast variety of commensal and pathogenic organisms [16,33]. More recently, attempts have been made to better simulate the complex mucosal epithelial environment in vivo. Hjelm et al. have developed a three-dimensional organotypic human vaginal epithelial cell model, which has been shown to exhibit physiologically relevant features such as the stratified squamous epithelium with microvilli, tight junctions, microfolds, and mucus [34]. This model was applied for screening microbicide compounds for safety and efficacy; however, it has not been utilized to study host-pathogen interactions. To characterize aspects of the multilayered growth and polarity of the uterine cervical epithelium, studies have cultured human and porcine ectocervical epithelium in air-liquid interface (ALI) [35,36,37]. The ALI conditions were achieved by exposing the apical side of vaginal epithelial cells to air supplemented with 5% CO_2_ and the basolateral side to the growth medium, mimicking studies conducted in tracheal and bronchial epithelia to understand the complex functions of the human airway [38,39,40]. However, no studies have focused on vaginotropic viruses such as HSV-2.

We designed the current study to determine if vaginal cells grown under ALI culture conditions would have distinct morphology and functions compared to LLI conditions. We used the Vk2/E6E7 (Vk2) cell line that was developed by immortalizing normal vaginal cells with a defective retroviral construct expressing human papilloma virus (HPV) type 16 E6 and E7 (LXSN-16/E6E7) [41]. HPV E6 and E7 viral oncogenes increase epithelial cell proliferation but do not lead to complete transformation of the cell line. These cells were non-tumorigenic and maintained a stable epithelial phenotype similar to the cells they were derived from [41]. Primary vaginal tissue is difficult to obtain and even more difficult to culture because the majority of the cells are terminally differentiated. Many studies use HeLa and HEC-1 cells, which are transformed cells and their similarity to primary cells is debatable. Since the advent of the HPV immortalized cell lines, they have become the mainstay for researchers examining host-pathogen interactions [41,42,43,44,45,46,47]. The Vk2 cell line has been extensively characterized and used in a wide variety of studies, ranging from basic studies examining innate responses to bacterial and viral pathogens to pre-clinical assessment of genital epithelium response to microbicides [27,47,48,49,50]. Vk2 cells have been shown to also express a variety of steroid hormone receptors and respond to estradiol, progesterone, and glucocorticoids [29]. Therefore, these Vk2 cells are a suitable model to examine hormone effects.

Our goal in the current study was to determine the differences in Vk2 cell growth, morphology, cytokeratin expression, and HSV-2 susceptibility between ALI and the conventional LLI. Furthermore, to accurately simulate the in vivo environment of the lower FRT, we examined the effects of female sex hormones and MPA on the vaginal cell functions.

## 2. Materials and Methods

### 2.1. Generating Air-Liquid Interface (ALI) and Liquid-Liquid Interface (LLI) Cultures of Vk2 Cell Line

**Figure 1:**
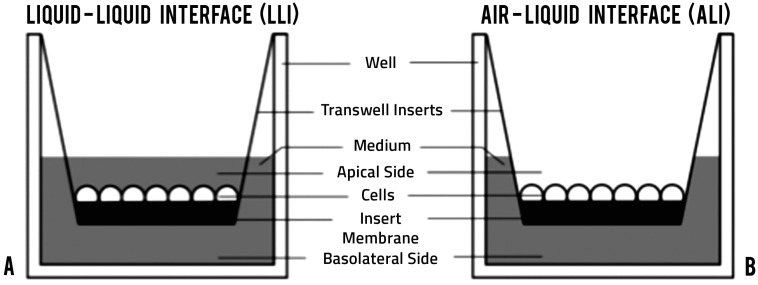
Culture design comparison. (**A**) liquid-liquid interface (LLI); (**B**) air-liquid interface (ALI).

To generate the Vk2/E6E7 (Vk2) culture, 60,000 cells were seeded on 0.4 µm pore-sized transwell polystyrene inserts (BD Falcon, Mississauga, ON, Canada), and keratinocyte serum-free medium (Life Technologies, Carlsbad, CA, USA) supplemented with 0.1 ng/mL human recombinant epidermal growth factor (Life Technologies), 0.05 mg/mL of bovine pituitary extract (Life Technologies), CaCl_2_, and 100 units/mL penicillin/streptomycin (Sigma Aldrich, Oakville, ON, Canada) were added to the apical and basolateral sides of the culture, as described in [41]. LLI culture was generated by keeping the media on both the apical (300 µL) and basolateral sides (500 µL) (Figure 1A). For ALI culture, media from the apical side was removed after 24 h of seeding the cells and media was only present in the basolateral side (1000 µL) (Figure 1B). Media was replenished every 24 h for 10 days of culture.

### 2.2. Hematoxylin and Eosin and Actin Staining of ALI and LLI Cultures

After three and 10 days of culture, Vk2 cells were fixed in 4% paraformaldehyde for 24 h and washed in phosphate-buffered saline (PBS; McMaster Immunology Research Centre Media Production Facility, Hamilton, ON, Canada). Cultures were then excised from transwell polystyrene inserts, stored in 70% ethanol, processed for histology and embedded in paraffin wax. Sections of 4–6 micron were cut and stained with hematoxylin and eosin (H&E) at the McMaster Immunology Research Centre Histology lab (Hamilton, ON, Canada). All samples were imaged on a Leica HC DMR microscope under 40× objective (Leica Microsystems, Wetzlar, Germany). To visualize the filamentous F-actin, after three and 10 days of culture, Vk2 cells were fixed in 4% paraformaldehyde, permeabilized with 0.1% Triton *X*-100 (Mallinckrodt Inc., Paris, KY, USA), and blocked for 30 min in blocking solution (5% bovine serum albumin and 5% goat serum (Sigma-Aldrich) in 0.1% Triton *X*-100 (Mallinckrodt Inc.)). Cultures were incubated with Texas Red-conjugated phalloidin (Sigma Aldrich) for 1 h at 37 °C. Cultures were then washed three times with PBS, and the polystyrene inserts were excised and mounted onto glass slides. Samples were imaged using an inverted laser-scanning confocal microscope at the McMaster Electron Microscopy Facility at 40× objective (LSM 510, Carl Zeiss Canada Ltd., Toronto, ON, Canada).

### 2.3. Pancytokeratin Staining of ALI and LLI Cultures Exposed to Female Sex Hormones and MPA

Estradiol (E2, Sigma Aldrich) (10^−9^ M), progesterone (P4, Sigma Aldrich) (10^−7^ M), or medroxyprogesterone acetate (MPA, Pfizer, Montreal, QC, Canada) (10^−9^ M) were added to the keratinocyte serum-free medium (Life Technologies) and Vk2 cells were grown in hormone-supplemented medium in the same volume as described above. Cells grown in medium containing different female sex hormones E2, P4, or MPA were cultured for 10 days, and fixed with 4% paraformaldehyde, washed with PBS, permeabilized with 0.1% Triton *X*-100 (Mallinckrodt Inc.), and blocked for 30 min in a blocking solution (5% bovine serum albumin and 5% goat serum (Sigma Aldrich) in 0.1% Triton *X*-100). To visualize human cytokeratin 10, 13, 18, and 19 that are present in Vk2 cells, monoclonal anti-cytokeratin pan antibody (Sigma Aldrich) was diluted to 1:200 in blocking solution and incubated with cultures for 1 h at 37 °C. Then, the cultures were washed with PBS and incubated with the secondary antibody, Alexa Fluor 488-conjugated goat anti-mouse IgG (1.5 μg/μL; Molecular Probes, Eugene, OR, USA), for 1 h at room temperature. Cell nuclei were stained with propidium iodide (500 nM; Molecular Probes). Cultures were then washed three times with PBS, and the polystyrene inserts were excised and mounted onto glass slides. Samples were imaged using an inverted laser-scanning confocal microscope at the McMaster Electron Microscopy Facility at 20× objective (LSM 510, Carl Zeiss Canada Ltd., Toronto, ON, Canada).

### 2.4. Cell Viability Assay

Vk2 cell viability under different hormonal conditions was assessed by trypan blue exclusion assay [51]. Vk2 cells exposed to no hormone (NH), E2, P4, or MPA were detached from the transwell inserts by gently scraping the cells with the pipette tip and were pelleted down, and re-suspended in 10% fetal bovine serum (FBS) Dulbecco’s modified Eagle’s medium (DMEM). Resuspended Vk2 cells were exposed to trypan blue dye (Life Technologies Inc.) in a 1:1 ratio of cell suspension to dye. Cells were loaded onto a hemocytometer and counted under a light microscope. Cell viability was assessed by subtracting the number of dead cells, stained in blue, from the total number of cells and was calculated in terms of percent of live cells.

### 2.5. HSV-2 Infection in LLI and ALI Culture Exposed to Female Sex Hormones and MPA

After 10 days of culture in medium containing different female sex hormones, Vk2 cells were infected apically with 10^4^ plaque-forming units (PFU) of wild-type HSV-2 or thymidine kinase (TK)^−^ HSV-2-green fluorescent protein (GFP) in 100 μL of respective keratinocyte serum-free hormone containing media (NH, E2, P4, or MPA). The cells were infected with HSV-2 for 2 h at 37 °C as previously described [52]. Briefly, after 2 h the inoculum was removed, cells were washed and the respective hormone media was added to both the apical (300 μL) and basolateral (500 μL) sides. The cells were incubated at 37 °C for 24 h and the supernatants were collected from the apical and basolateral sides and titered by plaque assay on Vero cells, as described before to measure viral shedding [19]. Briefly, Vero cells were grown to 80% confluence in 24-well plates. Samples were diluted (10^−2^ to 10^−7^) and added to monolayers. Infected monolayers were incubated at 37 °C for 2 h for viral adsorption. After 2 h, viral supernatants were removed and wells were replenished with media without any virus. Infection was allowed to occur for 48 h at 37 °C. Monolayers were then fixed and stained with crystal violet, and viral plaques were counted under a light microscope. PFU per milliliter was calculated by taking a plaque count for every sample and taking into account the dilution factor. For HSV-2-GFP infected cells, cultures were washed with PBS five times and the cell nuclei were stained with propidium iodide (500 nM; Molecular Probes). Cultures were then washed three times with PBS, excised from polystyrene inserts, and mounted onto glass slides. Samples were imaged using an EVOS™ FL digital inverted fluorescence microscope (Thermo Fisher Scientific, Burlington, ON, Canada) under a 20× objective.

### 2.6. Statistical Analysis

GraphPad Prism Version 5 (GraphPad Software, San Diego, CA, USA) was used to compare three or more means by one-way analysis of variance (ANOVA). When an overall statistically significant difference was seen, post-tests were performed to compare pairs of treatments, and the Bonferroni’s multiple comparison method was used to adjust the *p*-value. An alpha value of 0.05 was set for statistical significance. *p*-values for each analysis are indicated in the figure legends.

## 3. Results

### 3.1. Vk2 Cells Grow into Multi-Layered Cultures in ALI Conditions

**Figure 2:**
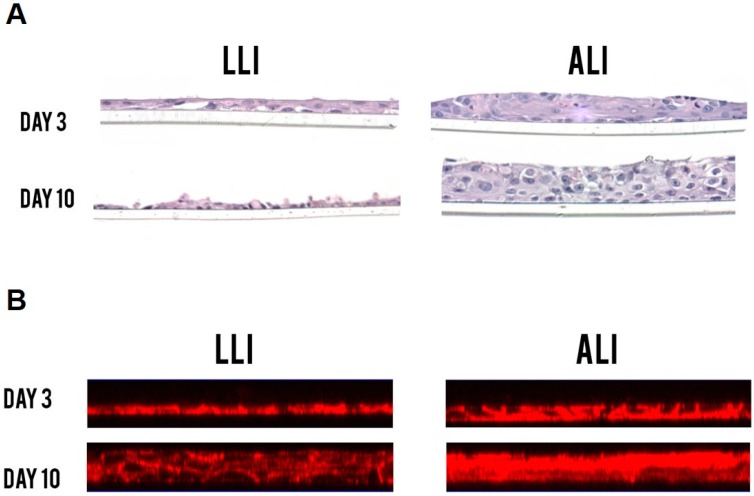
Hematoxylin and eosin (H&E) staining (**A**) and filamentous F-actin staining (**B**) comparison between the LLI and the ALI interface of Vk2 cells after 10 days of culture. Vk2 cells in ALI culture grew into multilayered epithelium compared to the monolayers seen in LLI. All samples were imaged on Leica HC DMR microscope under a 40× objective (Leica Microsystems). A minimum of three replicates per experimental condition was included. Representative images are shown.

To determine whether the ALI conditions lead to epithelial cell cultures that physiologically simulate in vivo vaginal cells compared to the LLI conditions, we first examined the cell growth and morphology in these cultures through H&E staining and F-actin staining. Confluent Vk2 cells were fixed and stained after three and 10 days of culture. Vk2 cells in LLI culture grew as monolayers of simple squamous epithelium, while the cells in ALI culture grew and differentiated into multilayered, stratified squamous epithelium, which is analogous to the vaginal morphology in vivo (Figure 2). These results provided evidence that ALI culture conditions resemble more closely the vaginal cells in vivo and could potentially be a more relevant model to study the vaginal cell interactions in the lower FRT.

### 3.2. Estradiol Increases Cytokeratin Expression

**Figure 3:**
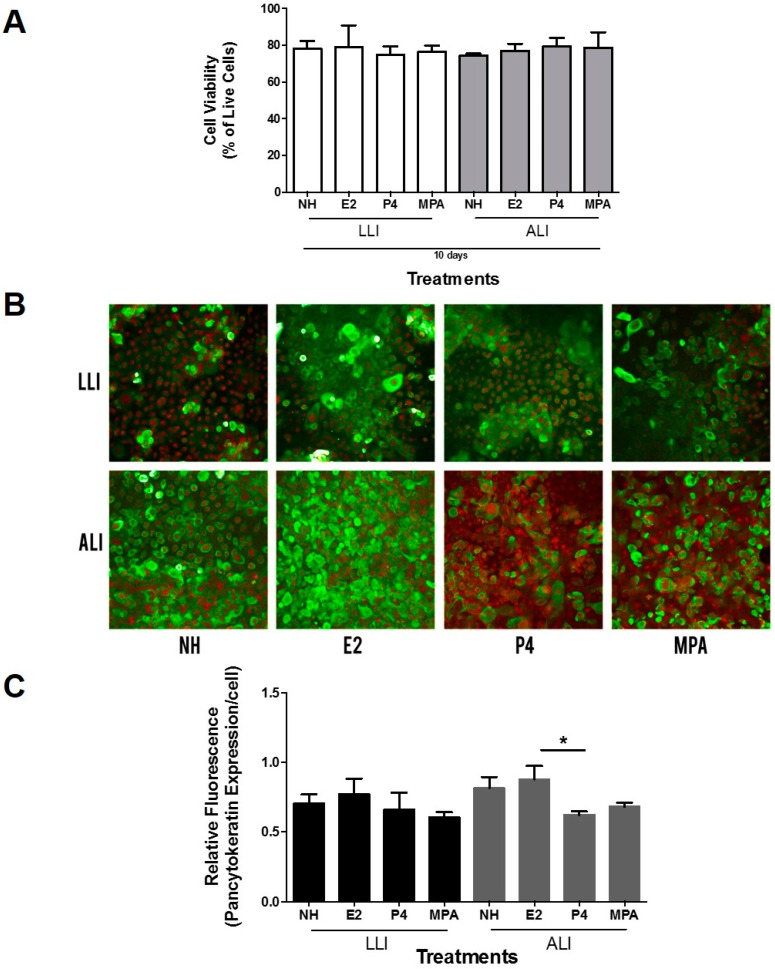
Cytokeratin staining comparison between the LLI and ALI of Vk2 cells after treatment with female sex hormones and medroxyprogesterone acetate (MPA). (**A**) Vk2 cells grown to confluence for 10 days were exposed to no hormone (NH), estradiol (E2; 10^−9^ M), progesterone (P4; 10^−7^ M), and medroxyprogesterone acetate (MPA; 10^−9^ M). Cell viability was measured using the trypan blue assay and expressed as percent of live cells in culture; (**B**) Pancytokeratin staining, green fluorescence, was done using mouse anti-human monoclonal anti-pan cytokeratin antibody. Propidium iodide was used for nuclear counter-staining, red fluorescence. All samples were imaged on an inverted confocal laser-scanning microscope. Representative images are shown. Images were taken in the *X*, *Y* plane; (**C**) Relative fluorescence of cytokeratin (green) to nuclear stain (red) was measured to quantify the amount of cytokeratin present per cell. Data shown represent mean ± standard error of the mean (SEM) of three separate cultures. * *p* < 0.05. Three replicates per experimental condition were included for each experiment. Magnification 20×.

Since we observed a clear difference in stratification patterns between ALI and LLI cultures, we next measured the ability of cells to express cytokeratin, an epithelial cell specific protein, to study the differentiation of Vk2 cells under these distinct conditions. Since vaginal cells and their functions, including growth and differentiation, are profoundly affected by female sex hormones, we decided to compare cytokeratin expression in the presence of E2, P4, or MPA. The hormone concentrations used for these studies were based on the serum concentrations of E2 and P4 seen under physiological conditions (menstrual cycle E2: 10^−11^ M to 10^−10^ M and P4: 10^−9^ M to 10^−8^ M; pregnancy E2: 10^−9^ M and P4: 10^−6^ M) and serum levels of Depot medroxyprogesterone acetate (DMPA) observed following subcutaneous injection (1 ng/mL) and have been described before [26]. Exposing Vk2 cells to different hormones over 10 days did not affect cell viability (Figure 3A).

Cytokeratins are proteins expressed in the cytoplasm of epithelial cells as part of a complex network that forms the cytoskeleton and extends from the surface of the nucleus to the cell membrane [53,54]. Additionally, they interact with desmosomes and hemidesmosomes, thereby assisting in cell-to-cell adhesion and barrier function [53,54,55]. Vk2 cells are known to express human cytokeratin 10, 13, 18, and 19 [41]. In addition, estradiol is known to upregulate vaginal epithelium proliferation and cytokeratin expression, as well as many other proteins found in epithelial cells [56,57,58,59,60]. The vaginal epithelium in women undergoes subtle yet statistically significant thickening during the menstrual cycle, unlike the very clear thickening seen in rodent species at estrus [59].

In order to determine if hormone treatments had an effect on cytokeratin expression under ALI and LLI conditions, Vk2 cells grown under different conditions were stained using a pan-cytokeratin antibody. Both ALI and LLI cultures appeared to show higher expression of cytokeratin proteins in E2 treated cells (Figure 3B, green fluorescence). Relative fluorescence of the images was measured using the ImageJ software and their ratio was calculated for each field. When the fluorescence was normalized to the nuclear staining, a significant difference in cytokeratin expression was observed between E2 and P4 treatment under ALI conditions (Figure 3C), possibly due to the increased proliferation of cells in E2 cultures. These results indicate that the effect of hormones on cytokeratin expression in vaginal cells was more pronounced under ALI conditions. 

### 3.3. HSV-2/GFP Infection in Vk2 Cells Cultured in Progesterone Is Higher Than in Estradiol

**Figure 4:**
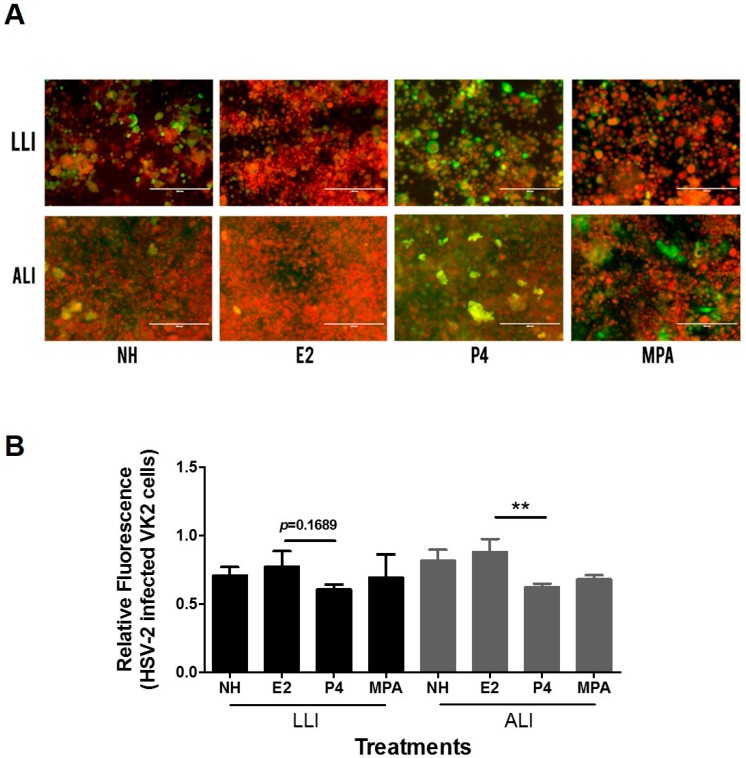
Effect of female sex hormones and MPA on HSV-2-GFP infection in Vk2 cells grown in ALI and LLI. Vk2 cells grown for 10 days to confluence in the presence or absence of female sex hormones were exposed to HSV-2-GFP at 10^4^ plaque-forming units (PFU) for two hours in both ALI and LLI. Nuclei were counter-stained with propidium iodide. (**A**) Images were taken to compare green fluorescence visually; (**B**) Relative fluorescence of HSV-2/GFP (green) to nuclear stain (red) was measured to quantify the amount of HSV-2 infection per cell. Data shown represented mean ± SEM of three separate cultures. ** *p* < 0.01. All samples were imaged on an EVOS™ FL digital inverted fluorescence microscope with a 20× objective. Images presented are representative of one of three images acquired from the experiments.

Since the effect of hormones on epithelial cell differentiation appeared to be enhanced in ALI conditions, we next examined the effects of female sex hormones on susceptibility to HSV-2 infection. We infected Vk2 cells grown in ALI and LLI conditions with a GFP-labelled thymidine kinase HSV-2 virus (HSV-2-GFP) and used fluorescence microscopy to visualize the infection. The relative fluorescence of the HSV-2-GFP (green) and the nuclear stain (red) was measured using the ImageJ software and their ratio was calculated in each field, in order to estimate the viral infection per cell. Under ALI conditions, Vk2 cells grown and exposed to HSV-2-GFP in the presence of P4 showed a significantly higher infection per cell compared to the E2 group (Figure 4A). A similar trend of higher relative fluorescence was seen in the P4 group in LLI cultures as well, but did not reach statistical significance. Overall, the highest infections were visualized in fluorescence microscopy images of the P4 conditions in both ALI and LLI (Figure 4B). These results indicate that P4 may play a role in increasing the susceptibility of Vk2 to HSV-2, compared to E2 treated cultures.

### 3.4. Progesterone Increases Viral Replication in Vk2 Cells in Both ALI and LLI Cultures

**Figure 5:**
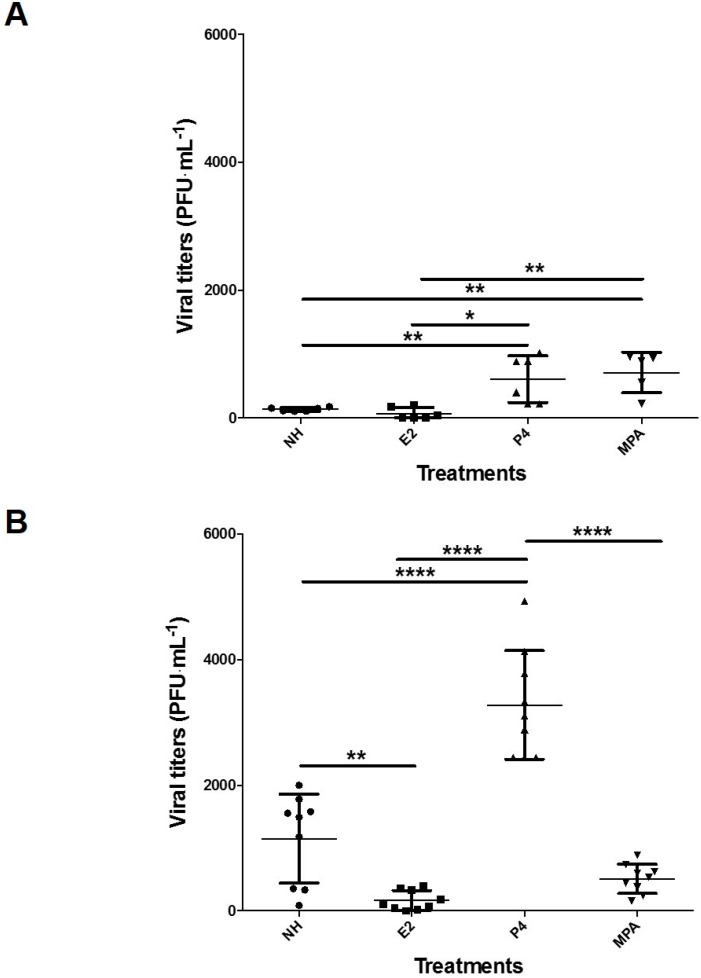
Difference in HSV-2 virus shedding by Vk2 cells in ALI and LLI conditions, exposed to different female sex hormones and MPA. To measure the viral shedding in Vk2 cell supernatants, Vk2 cells were exposed to 10^4^ PFU of wild-type HSV-2 for 2 h in both LLI (**A**) and ALI (**B**). Apical supernatants were collected and shed virus was measured by plaque assay on Vero cells (PFU/mL). Data were pooled from three separate experiments and are shown as mean ± SEM. * *p* < 0.05, ** *p* < 0.01, **** *p* < 0.0001.

To confirm the effect of P4 on the enhancement of HSV-2 infection, we measured the amount of virus shed by the Vk2 cells in ALI or LLI cultures, an indicator of virus replication, following each sex hormone treatment. Confluent Vk2 cultures exposed to sex hormones in ALI or LLI conditions were infected with wild-type HSV-2, and at 24 h post-infection, apical and basolateral cell culture supernatants were collected to measure viral shedding. Overall, Vk2 cells in the ALI culture showed higher viral shedding than in the LLI culture (Figure 5). In LLI, cells exposed to P4 and MPA showed significantly higher viral shedding than when exposed to E2 and the no hormone control (Figure 5A), while in ALI, P4 group showed significantly higher viral titers than all other treatment groups (*p* < 0.0001) (Figure 5B). Thus, the trend of the P4 group under ALI conditions displaying the highest infectivity and the E2 group displaying the lowest was consistent with the HSV-2-GFP infection results in ALI (Figure 4B). Overall there was consistency between the ALI and LLI cultures in that both showed significant increase in HSV-2 shedding in the P4 group compared to the control (no hormone) and E2. These results also suggest that E2 may play a protective role against HSV-2 infection.

## 4. Discussion

To the best of our knowledge, this is the first study that compared air-liquid interface to liquid-liquid interface cultures in order to examine under which conditions the vaginal Vk2 cells simulate in vivo growth and functions. Furthermore, we examined the effects of female sex hormones in Vk2 cell lines in terms of growth, differentiation, as well as HSV-2 infection under both ALI and LLI conditions. We demonstrated that ALI conditions support the development of stratified squamous Vk2 cell layers, which simulates the in vivo vaginal physiology more closely than LLI and therefore may be a better model to study host-pathogen interactions in the lower FRT. Vk2 cells showed a difference in susceptibility to HSV-2 under different hormonal conditions, particularly in ALI conditions. The presence of female sex hormones and MPA further enhanced the physiological relevance of the infection model as circulating hormones can alter the immune responses of the vaginal cells against the virus [17,18,19,20,21,22,23,24]. Based on these results, we conclude that ALI conditions represent more closely the FRT microenvironment and therefore may be a better model to study interactions between pathogens and the lower genital tract [15,16].

A significant change in susceptibility was noted between Vk2 cells treated with P4 compared to E2, especially in ALI cultures. The changes in susceptibility to HSV-2 under these conditions have never been reported. Our results demonstrate that P4 led to an increased HSV-2 infection and replication in Vk2 cells grown in ALI and LLI. While viral shedding was higher in both P4 and MPA under LLI conditions, P4 showed significantly higher viral replication in ALI cultures. This is consistent with many clinical and experimental studies showing that P4 can lead to increased susceptibility to sexually-transmitted infections (STI) like HSV-2, *Candida albicans*, and HIV-1 [19,58,61]. Marx et al. used subcutaneous hormonal implants in rhesus macaques and showed that P4 made the monkeys more susceptible to simian immunodeficiency virus type 1 (SIV-1) infection, while E2 demonstrated protective effects [62]. Therefore, the results from these studies, including our own, suggest that P4 levels can play a significant role in altering the female’s susceptibility to STIs. Although the mechanism of enhanced viral replication in P4-treated cultures is not clear, ALI conditions could trigger changes such as enhanced HSV-2 receptor expression or downregulation of certain antiviral innate responses. Further studies to better characterize the effects of E2 and P4 on susceptibility and innate responses to HSV-2 are warranted.

In vivo the vaginal squamous epithelium undergoes stratification naturally, such that the top layers are easily sloughed off and are continuously replaced by the underlying layers. While the superficial layers of vaginal epithelium undergo keratinization in some species, including mice, the human vaginal epithelium is not keratinized [59]. It does however show “cornification”, a process where the top layers are terminally differentiated and form the “stratum corneum” of the vaginal epithelium [12]. While the ALI cultures do show multiple layers, we have not extensively examined whether they became cornified. Future work may examine if such a conversion occurs in the top layers.

The use of ALI cultures in understanding the cellular responses between the host and the various commensal and pathogenic organisms in the female reproductive tract has great potential. Although our study only examined the interaction between the Vk2 cells and HSV-2, ALI conditions could be employed to culture other cell lines from the FRT such as ectocervical (ECT1/E6E7) and endocervical (END-1/E6E7) cell lines [41,42,43,46,55]. Additionally, ALI could be used to investigate the interactions between the host cells and other sexually transmitted pathogens such as hepatitis viruses, HIV-1, and *Chlamydia trachomatis*.

We believe that the ALI culture can be further improved to increase its physiological relevance. Vaginal bacterial communities, which are composed of over 265 microbial species, are thought to play an important role in preventing colonization by pathogenic organisms in the lower FRT [7,55,63]. Bacteria like *Lactobacillus* are known to contribute maintaining a healthy vagina and restricting the growth of foreign organisms by producing bacteriocins and lactic acid, as well as by acidifying the vaginal environment [63]. The addition of bacteria commonly found in the vaginal tract and of different female sex hormones to the ALI culture, could be used to further examine the Vk2 cell’s immune responses against STIs [43]. We limited our study to examining individual effects of E2 and P4, however, when present together, their interactions can be highly complex. Indeed, E2 and P4 coexist in vivo and their relative levels fluctuate throughout the reproductive cycle [4,15]. Hence, studies testing the susceptibility of Vk2 cells to HSV-2 in the presence of both E2 and P4 in varying concentrations are needed to examine their potential synergistic or antagonistic effects.

## 5. Conclusions

Overall, ALI cultures of Vk2 cells can be used in a broad range of reproductive immunology studies to understand how vaginal epithelial cells react in presence of viruses or other microbes in controlled and physiological conditions close to the FRT. Furthermore, our study provides an understanding that different hormonal conditions in the FRT can contribute to alter susceptibility towards STIs like HSV-2. Therefore, hormonal microenvironments should be carefully monitored when using P4-based contraceptives or other hormonal therapies to prevent a risk of higher susceptibility to STIs.
